# Associations between diagnostic pathways and care experience in colorectal cancer: evidence from patient-reported data

**DOI:** 10.1136/flgastro-2017-100926

**Published:** 2018-04-20

**Authors:** Theodosia Salika, Gary A Abel, Silvia C Mendonca, Christian von Wagner, Cristina Renzi, Annie Herbert, Sean McPhail, Georgios Lyratzopoulos

**Affiliations:** 1 Epidemiology of Cancer Healthcare and Outcomes (ECHO) Group, Department of Behavioural Science and Health, University College London, London, UK; 2 University of Exeter Medical School (Primary Care), Exeter, UK; 3 The Health Improvement Studies (THIS) Institute, University of Cambridge, Cambridge, UK; 4 National Cancer Registration and Analysis Service, Public Health England, London, UK

**Keywords:** colorectal neoplasm, cancer epidemiology, health service research, primary care, psychology

## Abstract

**Objective:**

To examine how different pathways to diagnosis of colorectal cancer may be associated with the experience of subsequent care.

**Design:**

Patient survey linked to information on diagnostic route.

English patients with colorectal cancer (analysis sample n=6837) who responded to a patient survey soon after their hospital treatment.

**Main outcome measures:**

Odds Ratios and adjusted proportions of negative evaluation of key aspects of care for colorectal cancer, including the experience of shared decision-making about treatment, specialist nursing and care coordination, by diagnostic route (ie, screening detection, emergency presentation, urgent and elective general practitioner referral).

**Results:**

For 14 of 18 questions, there was evidence (p≤0.02) for variation in patient experience by diagnostic route, with 6–31 percentage point differences between routes in adjusted proportions of negative experience. Emergency presenters were more likely to report a negative experience for most questions, including those about adequacy of information about their diagnosis and sufficient explanation before operations. Screen-detected patients were least likely to report negative experiences except for support from primary care. Patients diagnosed through elective primary care referrals were most likely to report worse experience for questions for which overall variation by route was generally small.

**Conclusions:**

Screening-detected patients tend to report the best and emergency presenters the worst experience of subsequent care. Improvement efforts can target care integration for screening-detected patients and provision of information about the diagnosis and treatment of emergency presenters.

## Introduction

Patient experience is increasingly regarded as a key outcome of cancer care. In England, the current national cancer strategy emphasises the importance of considering the care experience of patients with cancer ‘on a par with clinical effectiveness and safety’, as one of six national priorities for improvement.^1^ However, the predictors of positive or negative patient experience are poorly understood.

Different pathways to cancer diagnosis (otherwise known as ‘diagnostic routes’) are associated with variation in clinical outcomes.^2–4^ Because of the importance of early events in the cancer journey, different diagnostic routes may influence the experience of subsequent cancer care. This hypothesis is particularly applicable to colorectal cancer, which is characterised by large proportions of patients who are diagnosed through an emergency presentation and through screening, in addition to referred (primary-to-secondary care) routes. Understanding of associations between diagnostic routes and subsequent care experience can provide insights into predictors of negative patient experience and support the development of improvement interventions. However, detailed evidence about the presence, direction and size of associations between diagnostic pathways and experience of patients with cancer is lacking.

The English Cancer Patient Experience Survey (CPES) collects data on the experience of recently treated patients with cancer.^5^ Using data from this survey linked to information on diagnostic route, we aimed to identify how diagnostic pathways are associated with the experience of subsequent cancer care in patients with colorectal cancer.

## Methods

### Data

We analysed anonymous data on patients with colorectal cancer who responded to the Cancer Patient Experience Survey 2010—a postal survey of patients aged 16 or older who were treated for cancer in an National Health Service (NHS) hospital during January–March 2010.^6^ The survey was commissioned by the UK Department of Health and carried out by Quality Health, a specialist survey provider. A few weeks after the patients’ treatment and following relevant vital status checks, survey questionnaires were posted to patients (with up to two reminders to non-respondents); the response rate was 67%.^6^

We a priori restricted analyses to survey responders with a diagnosis of colorectal cancer (International Classification of Diseases-10 diagnosis codes C18–C20). We analysed data on patients with complete information on diagnostic route, based on data linkage with the Routes-to-Diagnosis data set. Routes-to-diagnosis denote different care pathways to the diagnosis of cancer (see the Exposure variables section below); they are algorithmically derived after linking cancer registration, Hospital Episode Statistics, screening and Cancer Waiting Times data sets.^4^ Linkage to CPES data was carried out previously by the Public Health England (former) National Cancer Intelligence Network to support public reporting of data on cancer patient experience as detailed previously.^7 8^

#### Outcomes

We a priori selected 18 survey questions representing major aspects of the patient journey from diagnosis to post-treatment care (see table 1). For questions with more than two response categories, we defined binary (positive/negative) experience outcomes, consistent with public reporting conventions from the CPES survey.^6 7^

**Table 1:** Patient survey questions on aspects of care experience in patients with colorectal cancer

Question number*	Synoptic form of question	Exact question wording
13	Told diagnosis sensitively	How do you feel about the way you were told you had cancer?
15	Written info on cancer diagnosis	When you were told you had cancer, were you given written information about the type of cancer you had?
18	Written info about treatment side-effects	Before you started your treatment, were you given written information about the side effects of treatment(s)?
19	Shared decision-making	Were you involved as much as you wanted to be in decisions about which treatment(s) you would have?
20	Given name of specialist nurse	Were you given the name of a clinical nurse specialist who would be in charge of your care?
21	Ease of contacting specialist nurse	How easy was it for you to contact your clinical nurse specialist?
30	Staff explained operation—before	Before you had your operation, did a member of staff explain what would be done during the operation?
32	Staff explained operation—after	After the operation, did a member of staff explain how it had gone in a way you could understand?
35	Confidence in hospital doctor	Did you have confidence and trust in the doctors treating you?
40	Confidence in ward nurse	Did you have confidence and trust in the ward nurses treating you?
43	Thought info withheld	While you were in hospital did you ever think that the doctors or nurses were deliberately not telling you certain things that you wanted to know?
49	Written info at discharge	Were you given clear written information about what you should or should not do after leaving hospital?
51	Self-management info post-discharge	Did the doctors or nurses give your family or someone close to you all the information they needed to help care for you at home?
58	Emotional support as out-patient	While you were being treated as an outpatient or day case, were you given enough emotional support from hospital staff?
60	Waiting time as out-patient	The last time you had an out-patient appointment with a cancer doctor at one of the hospitals named in the covering letter, how long after the stated appointment time did the appointment start?
63	Adequate info given to GP	As far as you know, was your GP given enough information about your condition and the treatment you had at the hospital?
64	General practice staff support	Do you think the GPs and nurses at your general practice did everything they could to support you while you were having cancer treatment?
65	Cancer care integration	Did the different people treating and caring for you (such as GP, hospital doctors, hospital nurses, specialist nurses, community nurses) work well together to give you the best possible care?

*Cancer Patient Experience Survey (2010).

GP, general practitioner

#### Exposure variables

Our main exposure variable was diagnostic route, comprising, for the purposes of this analysis, four routes as previously defined.^4^

- *Emergency presentation*: a diagnosis of cancer within 28 days from an emergency hospital admission or Accident and Emergency department attendance.
- *Screening detection:* as recorded in NHS Bowel Cancer Screening programme records.
- *Urgent referral for suspected cancer*: primary care referral for which patients have to be assessed by specialist hospital services within 2 weeks (hereafter denoted as ‘Two-Week-Wait’ (TWW) referral).
- *Elective primary care referral:* primary care referral other than TWW one; patients are assessed within routine outpatient appointments.

Patients with other (rarer) diagnostic routes were excluded from the analysis. Other exposure variables included patients’ sex, age (grouped as 16–44, 45–54, 55–64, 65–74, 75–84 and 85+), deprivation group (based on hospital records information included in the CPES dataset) and white/non-white ethnicity. We used self-reported ethnicity information (based on responses to a survey item) as it represents the gold standard for assigning ethnicity in routine data^9^; however, when self-reported information was missing (5%), we used ethnicity information as recorded by the hospital. Deprivation quintile groups were defined according to the Index of Multiple Deprivation 2007 scores of lower super output areas of residence, calculated using publicly reported cut-off values.^10^

### Analysis

For each of the 18 survey questions, we calculated the crude proportions of a negative experience by diagnosis route and subsequently used logistic regression to examine associations between diagnostic routes and patient experience. After calculating crude (unadjusted) Odds Ratios (ORs) of negative experience of care, we estimated ORs adjusted for sociodemographic variables (age, gender, ethnicity, deprivation quintile). Robust estimators of the standard error were used in regression models to account for potential clustering of observations within hospitals of treatment. To aid interpretation, using the outputs of the regression model used for multivariable analysis (above), we additionally estimated the adjusted proportions of negative experience by diagnostic route, assuming that the case mix of patients of each route was the same as that of the overall analysis sample. All analyses were carried out in STATA V.14.0.

## Results

**Figure 1:**
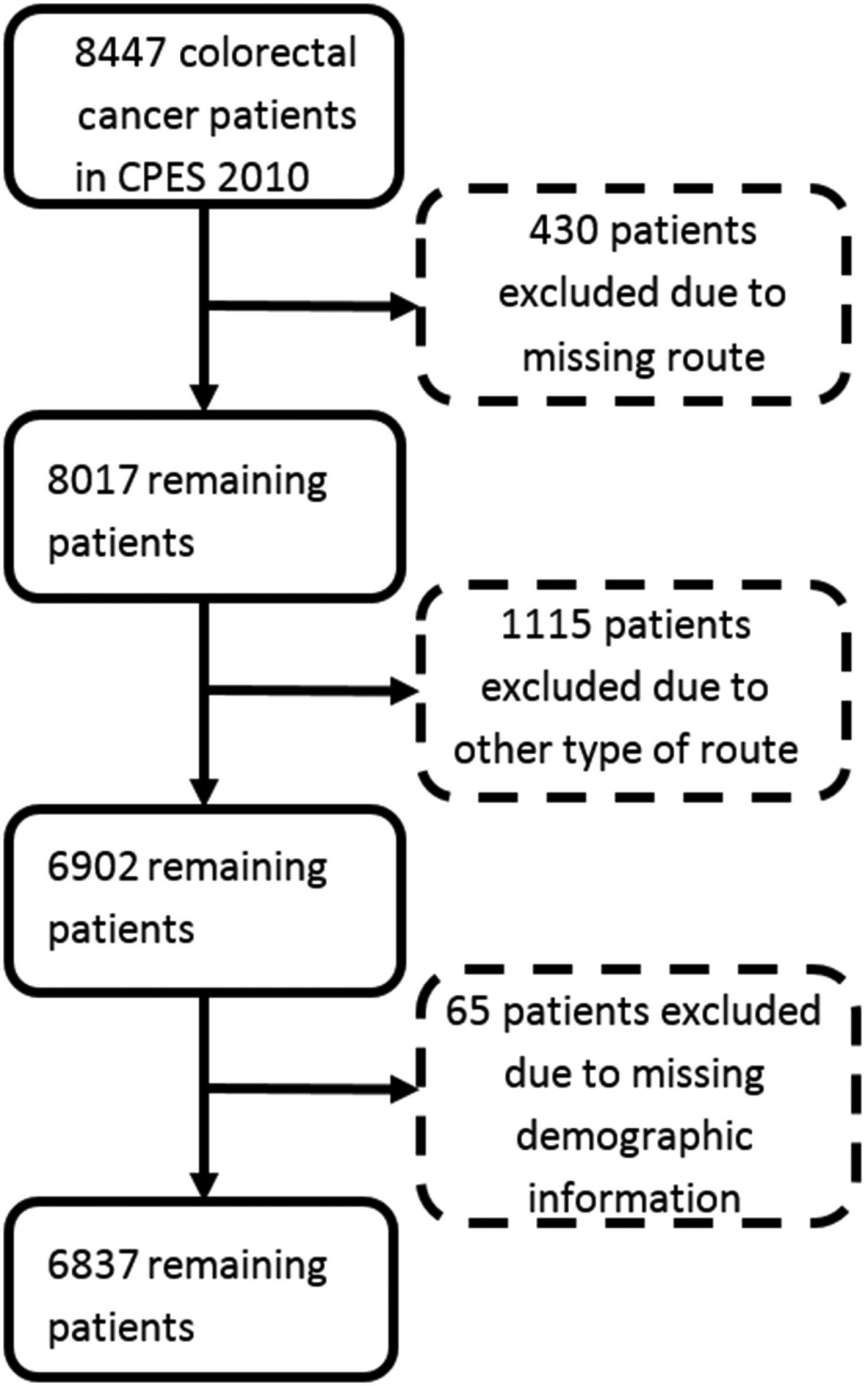
Analysis sample derivation.

After excluding cases with missing outcome or exposure variables, the analysis sample comprised 6837 patients with colorectal cancer (figure 1). Of those patients, 16%, 12%, 39% and 33% were diagnosed through an emergency presentation, screening, a TWW referral or an elective primary care referral, respectively. The observed proportion of patients reporting negative experience response options ranged from 6% (regarding information provided to the patient’s general practitioner) to 42% (regarding the provision of information to relatives to help care at home after discharge from hospital) (table 2).

**Figure 2:**
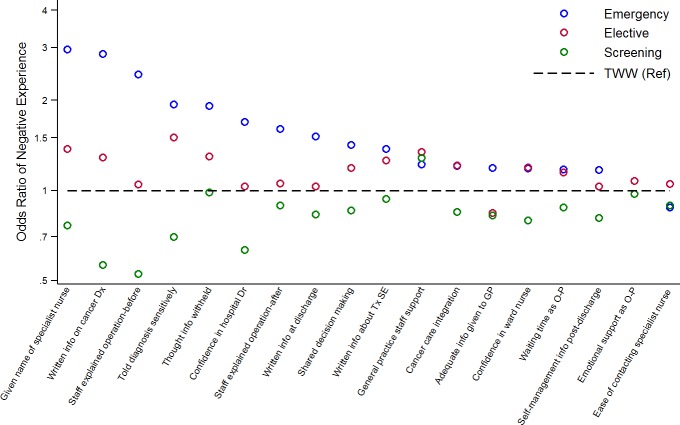
Adjusted odds ratios of negative experience by diagnostic route. Patients diagnosed through a Two-Week-Wait (TWW) route are the reference category. Estimates are shown only for the 14 (of 18) questions with statistical evidence for variation by route in our sample (p≤0.02). Dx, diagnosis; GP, general practitioner; O-P, outpatient; Ref, reference group; SE, side-effects; Tx, treatment.

Table 2 and figure 2 show the association between diagnostic routes and reported patient experience for the 18 studied items, adjusted for patient characteristics (unadjusted associations are shown in the online Supplementary appendices 1 and 2). Across all questions, screening-detected patients tend to be the ones least likely to report a negative experience, followed by those diagnosed via a TWW referral and those diagnosed via elective referral. Those diagnosed through an emergency presentation were generally most likely to report a negative experience.

10.1136/flgastro-2017-100926.supp1 Supplementary file 1

**Table 2:** Crude percentage and adjusted odds ratios of negative experience of care by studied survey question; questions appear in descending order of size of variation by diagnostic route (=penultimate column)

Question number	Synoptic form of question	N*	n	% Negative experience (crude) n/N	Adjusted ORs of negative experience by route†				Size of variation by route (max OR/min OR)	P values (for overall variation across the four studied routes)
					Emergency presentation	Elective referral	Two-Week-Wait referral (reference)	Screening detection		
15	Written info on cancer diagnosis	5610	1495	27	**2.85**	1.29		0.56	**5.09**	<0.0001
30	Staff explained operation—before	4944	802	16	**2.44**	1.04		0.53	**4.60**	<0.0001
20	Given name of specialist nurse	6348	651	10	**2.96**	1.38		0.76	**3.89**	<0.0001
13	Told diagnosis sensitively	6689	1025	15	**1.93**	1.50		0.70	**2.76**	<0.0001
35	Confidence in hospital doctor	5331	716	13	**1.69**	1.03		0.63	**2.68**	<0.0001
43	Thought info withheld	5312	710	13	**1.91**	1.30		0.98	**1.95**	<0.0001
49	Written info at discharge	4962	1050	21	**1.51**	1.03		0.83	**1.82**	<0.0001
32	Staff explained operation—after	4988	1134	23	**1.60**	1.05		0.89	**1.80**	<0.0001
19	Shared decision making	4899	1283	26	**1.41**	1.19	1	0.85	**1.66**	0.0001
40	Confidence in ward nurse	5326	1977	37	1.18	**1.19**		0.79	**1.51**	0.0001
18	Written info about treatment side effects	6080	795	13	**1.38**	1.26		0.93	**1.48**	0.0063
63	Information given to general practitioner	5243	328	6	**1.18**	0.84		0.82	1.44	0.17
51	Self-management info postdischarge family/others	4631	1942	42	**1.17**	1.03		0.81	1.44	0.20
65	Cancer care integration	6347	2389	38	1.20	**1.21**		0.85	**1.42**	0.0001
64	General practice staff support	4587	1337	29	1.22	**1.34**		1.28	**1.34**	0.0015
60	Waiting time as outpatient	5983	1755	29	**1.17**	1.15		0.87	**1.34**	0.012
21	Ease of contacting specialist nurse	5160	1120	22	0.88	**1.05**		0.89	1.18	0.29
58	Emotional support as outpatient	4529	1176	26	1.07	**1.08**		0.97	1.11	0.72

In columns 6-9, values shown in underlined fonts indicate the worst and those in bold fonts the best comparative experience across the four studied routes. In column 10 (size of overall variation between routes) bold fonts indicate questions with evidence p≤0.02).

*N varied by question, ranging from 4529 (emotional staff support as outpatient—Q58) to 6689 (told diagnosis sensitively—Q13) because some questions were not applicable to all patients.

†Adjusted for sex, age group, deprivation quintile, white/non-white ethnicity and colon/rectal subsite.

The size of variation in patient experience by diagnostic route differed substantially between the questions (maximum/minimum odds ratio values across diagnostic route groups varied between 1.1 and 5.1, table 2, penultimate column), with evidence (p<0.02) for variation in patient experience by diagnostic route for 14/18 questions. The questions with the largest variation in experience across routes included items about provision of written information about the diagnosis, explanation before and after an operation, the provision of a named specialist nurse and questions about interpersonal care aspects (table 2). Those questions where the above ordering of associations (screen detected, TWW, elective, emergency) was not observed were those associated with limited variation in patient experience by diagnostic route.

We also observe that across all questions, differences in patient experience between the TWW and the elective referral route were generally small. The single question where patients diagnosed via the TWW route were most likely to report negative experiences was a question about practice staff support (question 64).

Table 3 shows that for the 14 questions with statistical evidence of variation there was between a 6 and 31 percentage points difference in case-mix adjusted proportions of patient reporting negative experience.

## Discussion

### Summary of main findings

We identified variation in key aspects of patient experience by diagnostic route among patients with colorectal cancer. Asymptomatic detection (through screening) was typically associated with the best experience of subsequent cancer care. Emergency presentation was associated with substantially worse comparative experience of many aspects of care, including some relating to immediate management. Across questions, electively referred patients tend to rate their experience more negatively than TWW-referred patients, although such associations tend to be weak.

### Comparison with the literature

We know of no relevant peer-reviewed studies examining the impact of diagnostic routes on experience of patient with cancer. A recent in-depth review failed to identify any formal evidence from population-based studies about associations between emergency presentation and patient experience.^2^ A recent study in Danish patients who presented in primary care has indicated that those who were referred through a ‘fast-track’ pathway (similar to the TWW route in our study) tended to be more satisfied than those who were referred electively.^11^ A previous study using English cancer patient experience data indicated that a greater number of pre-referral consultations is associated with poorer experience of subsequent care, though this measure of diagnostic timeliness does not necessarily correspond to any specific diagnostic route.^12^

### Strengths and limitations

We used data from a large nationwide sample of colorectal patients with cancer, with information on diagnostic routes assigned using validated algorithms. Our analysis was adjusted for patient characteristics including sex, age and deprivation status, minimising concerns about potential confounding arising from known associations between these sociodemographic variables and diagnostic routes. We had no information on stage at diagnosis and treatment type, and therefore we were not able to explore the potential influence of these factors. The survey respondents are recently treated patients with cancer, not population-based incident cases. This may limit the generalisability of the findings, particularly regarding patients diagnosed through emergency presentation, who are under-represented among the survey responders. However, while we may, therefore, underestimate the overall prevalence of negative experience, such differences are unlikely to bias estimates of associations substantially.^13^ It is possible that, given the importance assigned by patients to timely diagnosis, some of the observed associations may reflect variation in diagnostic timeliness than diagnostic route.^14^ However, we had no data on the length of diagnostic intervals to examine this question empirically.

### Interpretation and implications for policy, practice and research

Emergency presentation was strongly associated with worse experience of subsequent care for the majority of the studied questions. Such associations were apparent both for questions relating to reports of actual processes of care (eg, whether a patient was given written information about the diagnosis) and the personal evaluation of the experience (eg, whether the patient felt they were told their diagnosis sensitively; see tables 2 and 3. These observations suggest that emergency presenters rate the same care processes differently, but also, often, experience different care processes. Most patients who are diagnosed with colorectal cancer in an emergency context will need emergency surgery, and clinical teams may tend to prioritise clinical as opposed to interpersonal care aspects in such circumstances. However, it should additionally be noted that differences are also apparent for questions relating to care processes expected to occur after the emergency context has abated, for example, access to specialist nursing or written information at discharge (see figure 2). Audit initiatives and further evidence about the immediate management of this patient group may help to identify opportunities for improvement.

**Table 3:** Adjusted percentage of negative experience

Question number	Synoptic form of question	Adjusted percentage of patients endorsing a negative experience				Absolute difference across routes (max adjusted %−min adjusted %)
		Emergency presentation	Elective referral	Two-Week-Wait referral	Screening detection	
15	Written info on cancer diagnosis	**45.4**	27.4	22.7	14.2	***31.2***
30	Staff explained operation—before	**29.8**	15.4	14.9	8.4	***21.4***
20	Given name of specialist nurse	**19.8**	10.3	7.7	6.0	***13.8***
13	Told diagnosis sensitively	**21.8**	17.8	12.7	9.3	***12.5***
35	Confidence in hospital doctor	**19.7**	13.1	12.8	8.5	***11.2***
43	Thought info withheld	**19.4**	14.0	11.2	11.0	***8.4***
49	Written info at discharge	**27.6**	20.7	20.3	17.5	***10.1***
32	Staff explained operation—after	**30.3**	22.3	21.4	19.5	***10.8***
19	Shared decision-making	**31.2**	27.6	24.4	21.6	***9.6***
40	Confidence in ward nurse	39.8	**40.0**	35.9	30.9	***9.1***
18	Written info about treatment side effects	15.3	14.2	11.8	11.1	*4.2*
63	Information given to general practitioner	7.6	5.6	6.5	5.5	*2.1*
51	Self-management info post-discharge family/others	45.5	42.4	41.8	36.9	***8.6***
65	Cancer care integration	40.2	**40.4**	36.0	32.3	***8.1***
64	General practice staff support	29.9	**31.9**	26.0	31.0	***5.9***
60	Waiting time as outpatient	**31.5**	31.1	28.2	25.6	***5.9***
21	Ease of contacting specialist nurse	19.8	**22.8**	22.0	20.1	*2.7*
58	Emotional support as outpatient	26.7	**26.7**	25.3	24.8	*1.9*

Bold/Italic fonts in the last column denote p≤0.02 for variation across all (four) routes (see also footnote of table 2).

In columns 3-6, underlined values denote the route associated with the worse experience and values in bold fonts the route associated with best experience.

Patients detected through screening reported best experience of care for all aspects of the patient journey that were studied, except for the item on ‘practice staff support’. This is explainable, as currently at least, there is no formal involvement of primary care in the English bowel cancer screening programme. It may be appropriate to consider a greater degree of integration of primary care in care pathways after screening detection.

Electively referred patients tended to rate their experiences more negatively than those diagnosed after a TWW referral. This may result from a sense of potential avoidable delay (among electively referred patients), consistent with previous evidence indicating that patients with cancer report better care experiences if they were referred promptly and through fast-track routes, given the importance of timely diagnosis for patients.^11 12 14^ It is also possible that patients referred onto the TWW care pathway experience more streamlined care, given that such pathways are designed to cater for patients in whom a cancer diagnosis is a priori suspected. Shortening of diagnostic intervals among patients diagnosed after an elective referral might also lead to improvements in care experience.

We conclude that decreasing the proportion of patients diagnosed through emergency presentation can be expected to improve the experience of cancer care. Similarly, increasing the proportion of patients who are screening-detected (eg, through increasing participation in colorectal cancer screening and reduction of related sociodemographic inequalities) could additionally result in improvements in patient-reported outcomes.^15 16^ Therefore, the findings provide additional impetus to efforts to reduce the frequency of diagnosis of colorectal cancer through emergency presentations and optimise participation in population-based bowel cancer-screening programmes. However, appreciable improvements in patient experience can be also achieved by efforts to improve the organisation and delivery of cancer care.

**Significance of this study**

**What is already known on this topic**

- We searched Medline for evidence examining associations between diagnostic pathways and patient-reported evaluations of subsequent experience of cancer care (aspects of this search formed part of an in-depth review on evidence about the predictors and consequences of diagnosis of cancer as an emergency (Zhou et al, reference 2). We found no studies examining this research question across emergency, screening and (fast-track or elective) referral routes to diagnosis of cancer.

**What this study adds**

- We document strong evidence for variation in key aspects of patient experience by diagnostic route among nearly 7000 patients with colorectal cancer. Screening detection was the diagnostic route associated with the most positive experience for nearly all studied questions. Emergency presentation was the diagnostic route associated with the most negative experience for most studied questions, including questions about immediate management.

**How might it impact on clinical practice in the foreseeable future**

- Improvements are needed in the management of emergency presenters with colorectal cancer, particularly regarding provision of information about their diagnosis and immediate management. Decreasing emergency presentations and increasing screening participation would improve both clinical outcomes and the care experience of patients with colorectal cancer.
