# Supplementary material for: Associations between diagnostic pathways and care experience in colorectal cancer: evidence from patient-reported data
Source: Frontline Gastroenterol. 2018 Apr 20;9(3):241–8. doi: 10.1136/flgastro-2017-100926 (PMC6056077; doi:10.1136/flgastro-2017-100926)
Supplement: Supplementary file 1 [file flgastro-2017-100926supp001.pdf]

Appendix 1. Crude and adjusted odds ratios (95% CIs) of negative experience compared to Two-Week-Wait referral group (bolded values represent 95% CI above/below parity)

			Crude odds ratios (95% CIs)				Adjusted odds ratios (95% CIs)			
Question Number	Question (synoptic form)	N	Emergency presentation	Elective referral	Two-Week-Wait referral (reference)	Screening detection	Emergency presentation	Elective referral	Two-Week-Wait referral (reference)	Screening detection
15	Written info on cancer Dx	5610	**2.77 (2.34, 3.28)**	**1.28 (1.11, 1.47)**		**0.52 (0.41, 0.65)**	**2.85 (2.40, 3.39)**	**1.29 (1.12, 1.49)**		**0.56 (0.44, 0.72)**
30	Staff explained operation-before	4944	**2.32 (1.89, 2.85)**	1.03 (0.86, 1.24)		**0.50 (0.37, 0.67)**	**2.44 (1.98, 3.02)**	1.04 (0.87, 1.26)		**0.53 (0.39, 0.71)**
20	Given name of specialist nurse	6348	**3.00 (2.42, 3.71)**	**1.38 (1.13, 1.69)**		0.73 (0.52, 1.01)	**2.96 (2.38, 3.69)**	**1.38 (1.12, 1.69)**		0.76 (0.54, 1.07)
13	Told diagnosis sensitively	6689	**1.92 (1.59, 2.31)**	**1.50 (1.28, 1.76)**		**0.73 (0.57, 0.95)**	**1.93 (1.59, 2.35)**	**1.50 (1.28, 1.76)**		**0.70 (0.54, 0.91)**
35	Confidence in hospital Dr	5331	**1.76 (1.42, 2.18)**	1.06 (0.88, 1.28)		**0.62 (0.46, 0.84)**	**1.69 (1.35, 2.11)**	1.03 (0.85, 1.25)		**0.63 (0.46, 0.86)**
43	Thought info withheld	5312	**1.91 (1.53, 2.38)**	**1.32 (1.09, 1.60)**		0.94 (0.71, 1.24)	**1.91 (1.53, 2.40)**	**1.30 (1.07, 1.57)**		0.98 (0.74, 1.30)
49	Written info at discharge	4962	**1.52 (1.25, 1.85)**	1.03 (0.88, 1.22)		0.80 (0.64, 1.01)	**1.51 (1.24, 1.85)**	1.03 (0.87, 1.22)		0.83 (0.65, 1.05)
32	Staff explained operation-after	4988	**1.59 (1.31, 1.92)**	1.06 (0.91, 1.25)		0.86 (0.69, 1.07)	**1.60 (1.32, 1.95)**	1.05 (0.90, 1.24)		0.89 (0.71, 1.11)
19	Shared decision making	4899	**1.35 (1.12, 1.62)**	**1.20 (1.03, 1.39)**	1	0.80 (0.64,1.00)	**1.41 (1.17, 1.72)**	**1.19 (1.02, 1.38)**	1	0.85 (0.68, 1.07)
40	Confidence in ward nurse	5326	1.17 (0.99, 1.38)	**1.19 (1.05, 1.36)**		**0.80 (0.67, 0.96)**	1.18 (1.00, 1.41)	**1.19 (1.04, 1.37)**		**0.79 (0.65, 0.96)**
18	Written info about Tx SE	6080	**1.31 (1.06, 1.62)**	**1.23 (1.03, 1.46)**		**0.67 (0.51, 0.89)**	**1.38 (1.10, 1.72)**	**1.26 (1.05, 1.51)**		0.93 (0.70, 1.25)
63	Information given to GP	5243	1.22 (0.89, 1.66)	0.88 (0.67, 1.15)		0.79 (0.53, 1.17)	1.18 (0.86, 1.63)	0.84 (0.64, 1.10)		0.82 (0.55, 1.24)
51	Self-management info post-discharge family/others	4631	**1.23 (1.03, 1.46)**	1.05 (0.91, 1.20)		**0.76 (0.63, 0.92)**	1.17 (0.97, 1.41)	1.03 (0.89, 1.19)		**0.81 (0.66, 0.99)**
65	Cancer care Integration	6347	**1.24 (1.07, 1.45)**	**1.24 (1.10, 1.39)**		**0.81 (0.68, 0.95)**	**1.20 (1.03, 1.41)**	**1.21 (1.07, 1.37)**		0.85 (0.71, 1.01)
64	General practice staff support	4587	**1.25 (1.03, 1.51)**	**1.37 (1.18, 1.59)**		1.20 (0.96, 1.50)	**1.22 (1.00, 1.48)**	**1.34 (1.15, 1.56)**		**1.28 (1.02, 1.62)**
60	Waiting time as O-P	5983	**1.19 (1.01, 1.40)**	**1.15 (1.01, 1.31)**		0.90 (0.75, 1.09)	1.17 (0.99, 1.39)	**1.15 (1.01, 1.31)**		0.87 (0.72, 1.06)
21	Ease of contacting specialist nurse	5160	0.91 (0.73, 1.12)	1.07 (0.91, 1.25)		0.91 (0.74, 1.13)	0.88 (0.70, 1.09)	1.05 (0.90, 1.23)		0.89 (0.71, 1.11)
58	Emotional support as O-P	4529	1.12 (0.93, 1.36)	1.11 (0.95, 1.30)		0.89 (0.71, 1.12)	1.07 (0.88, 1.31)	1.08 (0.92, 1.26)		0.97 (0.77, 1.23)

Appendix 2. Crude and adjusted % of negative experience.

Question Number	Question synoptic form	N	Emergency presentation	Elective referral	Two-Week-Wait referral	Screening detection	Emergency presentation	Elective referral	Two-Week-Wait referral	Screening detection
15	Written info on cancer Dx	5610	45.3	27.6	23.0	13.4	45.4	27.4	22.7	14.2
30	Staff explained operation-before	4944	29.2	15.5	15.1	8.1	29.8	15.4	14.9	8.4
20	Given name of specialist nurse	6348	20.1	10.4	7.7	5.8	19.8	10.3	7.7	6.0
13	Told diagnosis sensitively	6689	21.6	17.8	12.6	9.6	21.8	17.8	12.7	9.3
35	Confidence in hospital Dr	5331	20.3	13.3	12.6	8.2	19.7	13.1	12.8	8.5
43	Thought info withheld	5312	19.4	14.2	11.2	10.6	19.4	14.0	11.2	11.0
49	Written info at discharge	4962	27.9	20.8	20.3	16.9	27.6	20.7	20.3	17.5
32	Staff explained operation-after	4988	30.2	22.5	21.4	19.0	30.3	22.3	21.4	19.5
19	Shared decision making	4899	30.6	28.1	24.6	20.7	31.2	27.6	24.4	21.6
40	Confidence in ward nurse	5326	39.5	40.0	35.9	31.0	39.8	40.0	35.9	30.9
18	Written info about Tx SE	6080	15.5	14.6	12.3	8.6	15.3	14.	11.8	11.1
63	Information given to GP	5243	7.7	5.7	6.5	5.2	7.6	5.6	6.5	5.5
51	Self-management info post-discharge family/others	4631	46.7	42.8	41.6	35.1	45.5	42.4	41.8	36.9
65	Cancer care Integration	6347	41.0	40.8	35.8	31.0	40.2	40.4	36.0	32.3
64	General practice staff support	4587	30.4	32.3	25.9	29.5	29.9	31.9	26.0	31.0
60	Waiting time as O-P	5983	31.6	31.0	28.1	26.0	31.5	31.1	28.2	25.6
21	Ease of contacting specialist nurse	5160	20.1	22.9	21.8	20.3	19.8	22.8	22.0	20.1
58	Emotional support as O-P	4529	27.5	27.2	25.2	23.1	26.7	26.7	25.3	24.8
